# Odontogenic Tumors: A Challenge for Clinical Diagnosis and an Opportunity for AI Innovation

**DOI:** 10.30476/dentjods.2024.101237.2284

**Published:** 2024-06-01

**Authors:** Mohamad Reza Golzar Feshalami, Mehraban Shahi, Nasrin Davari Dolatabadi

**Affiliations:** 1 Student Research Committee, Faculty of Medicine, Hormozgan University of Medical Sciences, Bandar Abbas, Iran; 2 Dept. of Health Information Management, Dept. of Health Information Technology, School of Allied Medical Sciences, Hormozgan University of Medical Sciences, Bandar Abbas, Iran

## Dear Sir

The advancement of artificial intelligence (AI) has opened up new possibilities for medical diagnosis and treatment. In particular, AI algorithms have demonstrated remarkable potential in analyzing patient radiology images and histopathological samples, offering insights that can enhance clinical decision-making [ 1 ]. This letter explores the emerging role of AI in the diagnosis and treatment of odontogenic tumors (OTs), a group of benign, malignant, and tumor-like malformations arising from the remnants of the tooth-forming apparatus.

OTs are relatively rare, accounting for approximately 2.17% of all oral and maxillofacial tumors. They typically manifest in the third and fourth decades of life, presenting as destructive lesions of the jawbones [ 2 ]. The accurate diagnosis of OTs is crucial for determining appropriate treatment strategies and ensuring optimal patient outcomes.

Chai *et al*. [ 3 ] conducted a study using a convolutional neural network to differentiate between ameloblastoma and odontogenic keratocyst on cone-beam computed tomography (CBCT) images prior to surgery. The AI model demonstrated impressive sensitivity, specificity, accuracy, and F1 score of 87.2%, 82.1%, 84.6%, and 85.0%, respectively. In comparison, the diagnostic performance of 7 senior oral and maxillofacial surgeons and 30 junior surgeons showed lower accuracy, highlighting the potential of AI to outperform clinical expertise in differentiating these lesions.

Lee *et al*. [ 4 ] focused on the detection and diagnosis of three types of odontogenic cystic lesions, odontogenic keratocysts, dentigerous cysts, and periapical cysts using dental panoramic radiography and CBCT images. In their study, the deep convolutional neural network demonstrated excellent diagnostic performance, with an area under the curve of 0.914, sensitivity of 96.1%, and specificity of 77.1%. This model by using panoramic images showed the better performance, confirming the superiority of AI in the diagnosis of odontogenic cystic lesions [ 4 ].

The use of AI in dentistry goes beyond radiological diagnostics and includes a wide range of applications [ 5 - 7 ]. A study explored the use of deep learning to distinguish between ameloblastoma and ameloblastic carcinoma, two histologically similar OTs. The AI model, based on the ResNet50 architecture, achieved satisfactory performance, with average accuracy, precision, sensitivity, specificity, and F1-score of 0.75, 0.71, 0.84, 0.65, and 0.77, respectively. While the model demonstrated strong learning potential, it exhibited limited generalization ability due to the small data set [ 7 ].

The integration of AI within maxillofacial tumor management is intricately linked with medical imaging, bioinformatics, and medical robotics, which provide crucial data and tools for AI development and application [ 8 ]. AI-aided approaches, for example, enable more precise surgical resection of tumors and cysts, minimizing the need for additional procedures by providing surgeons with real-time insights into the anatomy and tumor location [ 9 ]. Additionally, computer-assisted surgery using preoperative simulation and augmented reality has emerged as a promising tool. This new augmented reality system eliminates the need for markers and uses image recognition of the teeth to overlay the surgical field with preoperative images. Moreover, it has the potential to provide surgeons with more accurate and efficient guidance during surgery, potentially leading to improved patient outcomes [ 10 ].

These findings demonstrate the potential of AI to improve the accuracy of OTs diagnosis, especially for less experienced surgeons. AI algorithms can provide objective and consistent assessments, reducing the risk of misinterpretation and delays in treatment initiation.

While AI has shown promising results in diagnosing OTs, its practical implementation faces technical, societal, and ethical limitations. These limitations can stem from data size, algorithm design, or clinical application challenges. Further research is necessary to explore AI's potential in other aspects of OT management, such as treatment planning, prognosis, and risk stratification.

Developing robust AI models requires large and diverse datasets of annotated images to ensure accurate and generalizable performance. However, concerns regarding data privacy, bias, and interpretability must be addressed to ensure responsible and ethical implementation.

Despite these challenges, AI holds immense potential for enhancing OTs diagnosis and treatment. As AI technology advances, its integration into oral medicine and maxillofacial surgery is expected to expand, leading to better patient outcomes and improved healthcare.
